# Diversity of immune cell types in multiple sclerosis and its animal model: Pathological and therapeutic implications

**DOI:** 10.1002/jnr.24023

**Published:** 2017-01-13

**Authors:** Yun Cheng, Li Sun, Zhongxiang Xie, Xueli Fan, Qingqing Cao, Jinming Han, Jie Zhu, Tao Jin

**Affiliations:** ^1^ Department of Neurology and Neuroscience Center First Hospital of Jilin University Changchun China; ^2^ Department of Neurobiology, Care Sciences and Society Karolinska Institute Stockholm Sweden

**Keywords:** multiple sclerosis, EAE, T cells, B cells, macrophage, tolerogenic dendritic cells, stem cells

## Abstract

Multiple sclerosis (MS) is an inflammatory, demyelinating disease of the central nervous system with an autoimmune attack on the components of the myelin sheath and axons. The etiology of the disease remains largely unknown, but it is commonly acknowledged that the development of MS probably results from the interaction of environmental factors in conjunction with a genetic predisposition. Current therapeutic approaches can only ameliorate the clinical symptoms or reduce the frequency of relapse in MS. Most drugs used in this disease broadly suppress the functions of immune effector cells, which can result in serious side effects. Thus, new therapeutic methods resulting in greater efficacy and lower toxicity are needed. Toward this end, cell‐based therapies are of increasing interest in the treatment of MS. Several immunoregulatory cell types, including regulatory T cells, regulatory B cells, M2 macrophages, tolerogenic dendritic cells, and stem cells, have been developed as novel therapeutic tools for the treatment of MS. In this Review, we summarize studies on the application of these cell populations for the treatment of MS and its animal model, experimental autoimmune encephalomyelitis, and call for further research on applications and mechanisms by which these cells act in the treatment of MS. © 2017 The Authors Journal of Neuroscience Research Published by Wiley Periodicals, Inc.

## INTRODUCTION

Multiple sclerosis (MS) is primarily a chronic inflammatory demyelinating disorder of the central nervous system (CNS) characterized by focal infiltration of lymphocytes and macrophages, and subsequent immune‐mediated damage to myelin and axons. The clinical onset of MS in patients usually manifests in their 20s and 30s and affects women about twice as often as men. While the etiologies in MS are hotly debated, the evidence obtained from animal models and patient studies indicated that abnormalities in the activity of different types of lymphocytes and the accompanying dysregulation of inflammatory cytokines play a crucial role in the pathogenesis of MS (Mastorodemos et al., 2015). So far, there has been no cure for MS. Experimental autoimmune encephalomyelitis (EAE) is a widely accepted animal model of MS that has been used to study the pathophysiology and therapy of MS. Currently available therapies for MS are aimed primarily at reducing the number of relapses and slowing the progression of disability. Conventional agents—including corticosteroids; recombinant interferon (IFN)‐β‐1a, 1b; glatiramer acetate; natalizumab; fingolimod; and others—are partially effective (Wingerchuk and Carter, 2014), but often result in serious side effects, such as infection, or secondary malignancy liking treatment‐related acute leukemia (Wingerchuk and Carter, 2014). Therefore, more safe and effective treatment plans need to be established. An improved understanding of the complexity of immune cells suggests that induction or delivery of specific cell types may offer promising and more tailored treatment of MS. Regulatory T cells (Tregs) with the strongest suppressive ability were found in the recovery phase of EAE (Koutrolos et al., 2014), and the lack or loss of regulatory B cells (Bregs) was shown to be associated with progression of MS (Knippenberg et al., 2011). Dendritic cells (DCs) are believed to be the main initiator of innate and adaptive immunity. They are important not only in the generation of T cell–mediated immune responses but also in the induction and maintenance of central and peripheral tolerance. Hematopoietic stem cell (HSC) transplantation potentially regenerates a new and more tolerant immune system and has begun to be considered by some as a curative therapy for MS. This article outlines the stem cell– and other cell–based therapies in MS and the technical difficulties and other challenges that need to be addressed prior to their general use.

## T CELL–BASED IMMUNOTHERAPY IN MS

MS is a chronic demyelinating inflammatory disease of the brain and spinal cord. The main pathological hallmarks of MS are the focal demyelination known as plaques, which consist of inflammatory cells, demyelination, reduced oligodendrocyte numbers, transected axons, and gliosis (Duffy et al., 2014). Currently, substantial discoveries have led to a generally accepted hypothesis that MS is mediated by activation of autoreactive myelin‐specific T cells that enter the CNS and initiate and/or propagate a chronic inflammatory response (Compston and Coles, 2008). EAE is an autoimmune disease in animal models of MS. It shares many clinical and pathological features with MS.

**Figure 1:**
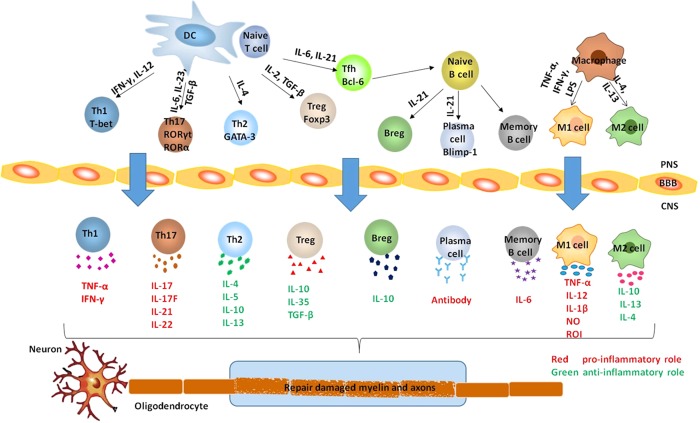
Pathogenesis of experimental autoimmune encephalomyelitis and multiple sclerosis. In the peripheral immune system, naive CD4 + T cells can differentiate into effector T helper cells depending on the cytokines, such as IL‐12, IL‐6, and TGF‐β, that secrete by APCs (macrophages, DCs, and B cells) together with costimulatory molecules (CD40, CD80, CD86) present on APCs. Effector T cells cross the BBB into the CNS. Activated T cells and macrophages with M1 type are proinflammatory and promote demyelination, axonal damage, and the formation of disease plaques, while macrophages with M2 type and Tregs have anti‐inflammatory, regulatory properties and inhibit disease progression by facilitating tissue repair. At the same time, with the help of Tfh cells, B cells differentiate into plasma cells and memory B cells. Plasma cells produce antibodies, which attack the myelin sheath on neurons. On the other hand, B cells promote both pathogenic and protective mechanisms by producing cytokines such as IL‐6 and IL‐10 in MS. APC indicates antigen‐presenting cell; BBB, blood‐brain barrier; Th, T helper; Treg, regulatory T; Tfh, follicular helper CD4+T; Breg, regulatory B; IL, interleukin; Bcl‐6, B cell lymphoma 6; Foxp3, forkhead box P3; GATA‐3, GATA‐binding protein 3; IFN‐γ, interferon‐γ; RORγt, retinoid‐related orphan receptor γt; RORα, retinoid‐related orphan receptor α; T‐bet, T‐box transcription factor; TGF‐β, transforming growth factor‐β; TNF‐α, tumor necrosis factor alpha; M1 cells, classically activated macrophages; M2 cells, alternatively activated macrophages; LPS, lipopolysaccharide; NO, nitric oxide; ROI, reactive oxygen intermediates. [Color figure can be viewed at wileyonlinelibrary.com]

For a long time, T cells have been at the center of research in MS immunology (Fig. 1). The differentiation of T helper (Th) cells is initiated by the combined signals mediated downstream of the T cell receptor (TCR) and cytokine receptors. Those signals then activate specific transcription factors responsible for the expression of lineage‐specific genes. Naive Th cells differentiate into Th1 cells when they are induced to express the transcription factor T‐bet, which occurs upon exposure to IFN‐γ and interleukin (IL)‐12 (Lazarevic et al., 2013). While in the presence of IL‐4, naive Th cells express the transcription factor GATA‐binding protein (GATA)‐3 and differentiate into Th2 cells (Meka et al., 2015). Th1 cells, which secrete IFN‐γ and tumor necrosis factor alpha (TNF‐α), were presumed by many to be the principal mediator of MS development (O'Brien et al., 2010). This conclusion was based on the observation that immunization with myelin antigens for EAE induction resulted in a domination of Th1 cells and abundant IFN‐γ in the CNS of animals with EAE. In addition, encephalitogenic Th1 cells were capable of inducing EAE when adoptively transferred into naïve recipient mice. Furthermore, mice deficient in T‐bet^−/−^ or signal transducer and activator of transcription (STAT)‐4 were resistant to EAE. STAT‐4 is a transcription factor necessary for IL‐12 signaling downstream of its receptor, and IL‐12 is required for differentiation of Th1 cells. Taken together, these findings led to the conclusion that Th1 cells play a critical role in EAE pathogenesis, and likely, by extension, also MS (Rostami and Ciric, 2013). However, several groups demonstrated that mice with genetic ablation of Th1 signature cytokine IFN‐γ or its receptor, as well as the IL‐12 subunit p35 or its receptor IL‐12Rβ2, were not only still susceptible to EAE but in some cases even developed more severe disease (Ferber et al., 1996; Willenborg et al., 1996; Becher et al., 2002; Gran et al., 2002; Zhang et al., 2003). The data questioned the Th1 paradigm, and this remained unexplained until the discovery of the Th17 cells. On the other end of the spectrum, Th2 cells, which secrete IL‐4, IL‐5, IL‐10, and IL‐13, have been proposed to provide protection from autoimmune response and neuronal damage in MS. Fernando et al. (2014) established novel transgenic (tg) mice that overexpress GATA3 and, thus, are Th2 biased. Compared with wild‐type mice, the GATA3‐tg mice had a significantly delayed onset and reduced severity of EAE.

The Th1 paradigm changed with the discovery of Th17 cells, which are characterized by producing IL‐17A (IL‐17), IL‐17F, IL‐21, and IL‐22 (Korn et al., 2009). Their main lineage‐specific transcription factor is the retinoic acid receptor–related orphan receptor γt (ROR‐γt) (Ivanov et al., 2006). However, ROR‐γt deficiency did not completely abolish the cytokine expression of Th17 cells. Together with ROR‐α, induced by transforming growth factor‐β (TGF‐β) and IL‐6 in a STAT‐3–dependent manner, ROR‐γt led to greater Th17 cell differentiation and activity (Yang et al., 2008). TGF‐β in combination with IL‐6 is responsible for the differentiation of naive CD4^+^ T cells into Th17 cells, a process amplified by autocrine production of IL‐21 (Sie et al., 2014). The cytokine IL‐23 is also critical for the inflammatory potential of Th17 cells, and an important survival factor for Th17 cells (Korn et al., 2009). In the absence of TGF‐β, a combination of IL‐1β, IL‐6, and IL‐23 induces differentiation of Th17 cells and promote the pathogenesis of EAE (Ghoreschi et al., 2010). Blockade of TGF‐β or IL‐23 signaling markedly suppresses Th17 differentiation and ameliorates EAE progression (Veldhoen et al., 2006; McGeachy et al., 2009). IL‐17 and IL‐17F belong to the IL‐17 family, which includes IL‐17, IL‐17B, IL‐17C, IL‐17D, IL‐17E, and IL‐17F (Korn et al., 2009). These cytokines induce several proinflammatory cytokines and chemokines, promote cellular infiltration, and have a strong proinflammatory influence on a series of cells (Mesquita et al., 2009). IL‐17 is the prototypic cytokine of the IL‐17 family, which is structurally homologous to the cysteine knot family of proteins (Petermann and Korn, 2011). IL‐17 has distinct proinflammatory functions (Liang et al., 2007). In patients with MS, the serum levels of IL‐17 and IL‐17F were higher, and it has also been shown that high expression of IL‐17 correlates with MS severity (Babaloo et al., 2015). Neutralization of IL‐17 or IL‐17 deficiency rendered mice resistant to the induction of EAE (Babaloo et al., 2015). These studies indicate that Th17 cells play a key role in the pathogenesis of EAE.

However, in recent years, it has been shown that Tregs take part in the immunopathogenesis of the disease as well. Tregs are known to limit the inflammatory reactions using different mechanisms, including direct inhibition of autoreactive T cell activation by secreting immunosuppressive mediators or cell‐to‐cell contact, or indirectly via inhibition of the stimulatory capacity of antigen‐presenting cells (APCs) (Schmidt et al., 2012). They are characterized by surface CD4 and CD25 expression and the transcription factor forkhead box P3 (Foxp3), which is essential for phenotypic and functional development of this cell lineage. They produce immunosuppressive cytokines, including IL‐10, IL‐35, and TGF‐β (Buc, 2013). IL‐2 and TGF‐β have been reported as crucial to induce the differentiation of naive CD4^+^ T cells into Treg cells (Kleinewietfeld and Hafler, 2013). Previous studies have indicated that Tregs suppress the Th1 and Th17 cell populations via production of their hallmark cytokines, such as IL‐10, IL‐35, and TGF‐β (Abdolahi et al., 2015). In relapsing–remitting EAE, depletion of Tregs increases acute‐phase severity, as well as preventing remission (Gartner et al., 2006; Zhang et al., 2006). The relative abundance of Tregs in patients with MS is unchanged when compared with controls, but their function measured in vitro may be diminished, correlating with impaired inhibitory activity in vivo (Costantino et al., 2008).

Owing to the key role of T cells in MS and EAE, there has been a vast amount of research in an effort to elucidate T cell–based immunotherapy. It is assumed that defects of central and peripheral tolerance permit an existence of self‐reactive T cells that lead to MS and EAE. To eliminate self‐reactive T cells and treat MS, it is important to induce and amplify potent physiological mechanisms of tolerance. Although several mechanisms, such as clonal deletion (the elimination of antigen‐specific cells by apoptosis) and clonal anergy (the induction of functional hyporesponsiveness to antigens), have all been suggested as mechanisms responsible for the immune unresponsiveness to self‐antigens, there is also substantial evidence that T cell–mediated active suppression of self‐reactive T cells by suppressor or Tregs is another essential mechanism of self‐tolerance in the periphery (Zhou et al., 2011). The results of adoptively transferred Tregs to EAE mice suggested that Tregs are highly potent suppressors of autoimmune EAE (Selvaraj and Geiger 2008). However, there are many challenges related to the use of Tregs in the treatment of MS. More studies are needed before Tregs can be clinically applied in MS.

## B CELL–BASED IMMUNOTHERAPY IN MS

Even though T cells are widely believed to play the central role in the pathogenesis of MS, there is also evidence supporting a pathogenic role of B cells. Most patients with MS show immunoglobulin G oligoclonal bands (OCBs) in their cerebrospinal fluid (CSF). OCBs are among the biomarkers used clinically for the diagnosis of MS (Compston and Coles, 2008; Abraira et al., 2011). In the presence of IL‐21 and IL‐6, naive T cells express transcription factor B cell lymphoma 6 and differentiate into follicular helper CD4^+^T cells, which provide help for B cell differentiation into plasma cells and memory B cells (Fan et al., 2015) (Fig. 1). IL‐21 also induces expression of Blimp‐1, a transcription factor that is critical to the differentiation of B cells into plasma cells (Gharibi et al., 2016). After differentiating to plasma cells, they produce autoantibodies. Histopathological studies have demonstrated that autoantibodies specific for myelin sheath protein, such as myelin basic protein autoantibodies, proteolipid protein autoantibodies, and myelin oligodendrocyte glycoprotein (MOG) autoantibodies, are associated with demyelination in EAE and MS (Lalive, 2008). MOG has been extensively studied as a target for autoantibodies in MS because it is selectively expressed by oligodendrocytes in the CNS. The role of anti‐MOG antibodies in patients with MS, however, has been controversial. Higher levels of antibodies to MOG have been reported in the serum and CSF of patients with MS than in controls (Kennel De March et al., 2003), but other studies have not observed any differences between the serum of patients with MS and controls or patients with other neurological diseases (Karni et al., 1999; O'Connor et al., 2005; Kuhle et al., 2007; Klawiter et al., 2010). Apart from their ability to secrete antibodies, activated B cells can also secrete cytokines, which influence T cell proliferation in patients with MS (Bar‐Or et al., 2010). B cells from patients with MS and EAE mice secreted more IL‐6 than B cells from controls. In addition, mice with a B cell–specific IL‐6 deficiency presented less severe disease than did mice with wild‐type B cells (Barr et al., 2012). This suggested that B cells can exert pathogenic effects in EAE and MS via secretion of IL‐6. Despite this, a population of activated B cells (Bregs) can exert regulatory, anti‐inflammatory actions via secretion of IL‐10 (Mauri and Bosma, 2012; Han et al., 2016). IL‐10 can inhibit production of a number of proinflammatory cytokines, including IL‐1β, IL‐6, IL‐12, GM‐CSF, and TNF‐α (Ireland et al., 2015). Moreover, IL‐6 supports Treg differentiation (Mauri and Bosma, 2012). The generation of Bregs in mice has been shown to depend in particular on the presence of IL‐21 and T cell interaction via CD40/CD40L (Yoshizaki et al., 2012). However, the identification of a Breg cell–specific transcription factor, similar to Foxp3 in Tregs cells, is still unknown.

Based on animal studies, B cells have the capacity to promote both pathogenic and protective mechanisms in MS. Although the exact role of B cells in MS remains unknown, clinical trials that target B cells have shed some light on the possible immunological mechanisms leading to more diverse and personalized treatment options for patients with MS. A variety of antibody treatments have been proposed to target B cells (Table 1). Rituximab, ocrelizumab, and ofatumumab are different anti‐CD20 depleting agents. CD20 is expressed on B cell subsets ranging from the pro–B cell stage of development in the bone marrow to mature circulating B cells in the periphery, but not on plasma cells (Krumbholz et al., 2012). In phase 2 clinical trials in relapsing–remitting patients with MS, rituximab significantly reduced the number of gadolinium‐enhancing lesions for 48 weeks compared with placebo controls (Hauser et al., 2008). In a phase 2 study of ocrelizumab for MS treatment, at week 24, the number of gadolinium‐enhancing lesions was lower in the ocrelizumab group than in the placebo group (Kappos et al., 2011). Ofatumumab is also used for treating MS (Sorensen et al., 2014). Among these monoclonal antibodies, rituximab is the most prominent one. The binding of rituximab to CD20 leads to the depletion of B cells via three different mechanisms: complement‐dependent cytotoxicity (CDC), antibody‐dependent cell‐mediated cytotoxicity (ADCC), and induction of B cell apoptosis (Gasperi et al., 2016). MEDI‐551, the monoclonal antibodies targeting CD19 on B cells, also show a promising new approach for depleting B cells (Herbst et al., 2010). Alemtuzumab is a humanized depleting monoclonal antibody that targets CD52, which is expressed on B cells, T cells, and monocytes and in the male reproductive tract (Krumbholz et al., 2012). It has been recently approved for the treatment of active MS with a direct depleting effect on both B and T cells (Cohen et al., 2012; Coles et al., 2012). Alemtuzumab depletes B cells, T cells, and monocytes by mechanisms of ADCC and CDC. Following depletion, B cells, monocytes, and T cells reconstitute homeostatic in the next few months. This leads to prolonged alteration of the immune repertoire (Milo, 2016). Despite these encouraging clinical trial results, the mechanism by which B cell depletion might be effective for MS treatment is still unknown. Insight into these mechanisms will provide a new approach for treating MS.

**Table 1:** Biological Drugs Targeting B Cells

Biologic	Species isotype	Target	Reference
Rituximab	Chimeric (murine/human) monoclonal IgG1	CD20	Hauser et al., 2008
Ocrelizumab	Humanized monoclonal IgG1	CD20	Kappos et al., 2011
Ofatumumab	Human monoclonal IgG1	CD20	Sorensen et al., 2014
MEDI‐551	Humanized monoclonal IgG1	CD19	Herbst et al., 2010
Alemtuzumab	Humanized monoclonal IgG1	CD52	Krumbholz et al., 2012; Cohen et al., 2012; Coles et al., 2012

## MACROPHAGES—THERAPY IN MS

In addition to lymphocytes, macrophages are also abundantly present in inflammatory MS lesions (Lucchinetti et al., 2000) (Fig. 1). Recent reports indicate that macrophages play dual roles in the pathogenesis of MS as they contribute to lesion formation and axonal damage, but also present repair mechanisms through the production of neurotrophic factors and anti‐inflammatory molecules as well as clearance of myelin debris (Abdul‐Majid et al., 2002; Kigerl et al., 2009).

There are dual origins of CNS macrophages, resident microglia and monocyte‐derived macrophages. Resident microglia originate from primitive macrophages that were established prior to birth. These cells arise from yolk sac in embryonic development and subsequently maintain the capacity for self‐renewing. Monocyte‐derived macrophages derive from monocytes that develop from bone marrow HSCs (Shemer and Jung, 2015). Depending on the tissue environment and the conditions that the cells encounter, macrophages can activate to two distinct phenotypes, with distinct “classically activated macrophages” (M1 cells) and “alternatively activated macrophages” (M2 cells) patterns. M1 cells are generally instigated by the presence of Th1 cytokines TNF‐α and IFN‐γ as well as microbial products, such as lipopolysaccharide (LPS). In contrast, M2 cells are activated by Th2 cell cytokines IL‐4 and IL‐13, release anti‐inflammatory cytokines, and promote tissue repair and fibrosis (McWhorter et al., 2015; Zhu et al., 2015). In EAE, M1 cells are associated with increased EAE severity, whilst M2 cells are correlated with ameliorated clinical disease (Porcheray et al., 2005). M1 cells upregulate CD86, CD40, and MHC II on their surface and are involved with T cell priming and recruitment into the CNS (Jiang et al., 2014). In addition, M1 cells secrete high levels of proinflammatory cytokines, such as TNF‐α, IL‐1β, IL‐12, nitric oxide, and reactive oxygen intermediates (Jiang et al., 2014). On the contrary, M2 cells promote the differentiation of Th2 and Tregs, which can suppress EAE severity (Weber et al., 2007). M2 cells produce anti‐inflammatory cytokines, such as IL‐10, IL‐13, and IL‐4 (Jiang et al., 2014). Adoptive transfer of M2 cells inhibited the development of Th17 cells and induced the differentiation of Th2 and Tregs, which could efficiently suppress EAE (Weber et al., 2007; Burger et al., 2009). Vogel et al. (2013) found that although macrophages in active MS lesions predominantly display M1 characteristics, the major subset of macrophages display an intermediate activation status, with coexpression of M1 and M2 markers. Therefore, shifting the phenotype of macrophages into the beneficial phenotype is an attractive therapy for EAE and MS.

## DC‐CELL BASED THERAPY IN MS

Although autoreactive T cells and their specificity for CNS antigens have been a major focus on MS research for some time, the generation of these cells depends on interactions with APCs. APCs process and present antigen and also express major histocompatibility complex (MHC) molecules for recognition by T cells via TCR. In the context of appropriate secondary signals, APCs can lead these antigen‐specific T cells toward particular effector profiles, categorized into Th1, Th2, Th17, or Tregs. DCs are a specialized subset of APCs. They play a pivotal role in the innate and adaptive immune systems. Under steady‐state conditions, the DCs within the peripheral tissues are in an immature phenotype, scanning self and foreign antigens, continuously capturing antigenic material, but lacking the ability to efficiently process and present antigens to T cells. Immature DCs may be activated by antigens, including self‐antigens, invading pathogens, and certain malignant cells, leading to mature DCs and activating T cells in lymphoid organs (Torres‐Aguilar et al., 2010). During this process, DCs notably increase the expression of MHC II (“signal 1”) and costimulatory molecules (“signal 2,” e.g., CD80, CD86, and CD40), secrete a wide variety of proinflammatory and anti‐inflammatory cytokines (“signal 3,” e.g., IL‐12, IL‐6, IL‐10, and TNF‐α), reduce their ability to take up antigens, and significantly augment their ability to stimulate T cells and increase their immunogenicity (Torres‐Aguilar et al., 2010; Chung et al., 2013).

DCs are classified into two major subsets, plasmacytoid DCs (pDCs) and conventional or classical DCs (cDCs), which show different morphological and functional characteristics (Quintana et al., 2015). Both subsets can display abnormalities during the course of MS. High numbers of cDCs and pDCs accumulate in the CSF and white matter of the CNS in patients with MS (Lande et al., 2008; Longhini et al., 2011), and it is generally accepted that DCs are involved in the pathogenesis of MS (Ganguly et al., 2013). For example, their increase in early disease and presence within lesions suggest that they participate in the pathophysiology of MS actively (Wu and Laufer, 2007). cDCs present a characteristic dendritic morphology and are endowed with potent APC function. cDCs are phagocytic cells that express high levels of MHC II (Quintana et al., 2015). cDCs from patients with MS showed increased expression of CD80 and CD40, and decreased programmed death ligand‐1 (Mackern‐Oberti et al., 2015). Meanwhile, cDCs from patients with MS produced more IL‐12 and TNF‐α compared with healthy controls (Mackern‐Oberti et al., 2015). On the other hand, pDCs are known to produce large amounts of type I interferon and other proinflammatory cytokines, contributing to protective immunity against viral and bacterial infections (von Glehn et al., 2012). pDCs are present in the CSF, and their concentration is increased in the CSF of patients with MS during an exacerbation (Longhini et al., 2011). pDCs promote activation of autoimmune Th17 cells in the early phase of EAE, whereas depletion of pDCs before induction of EAE decreases its severity (Isaksson et al., 2009). This suggests that DCs (cDCs in particular) from patients with MS exhibit a proinflammatory profile.

Although DCs play a crucial role at initiating immune response, they also contribute to maintaining peripheral immune tolerance. DCs mediate peripheral tolerance through numerous potential mechanisms—for example, by including apoptosis or deletion of mature autoreactive T cells (Chen et al., 2006), induced unresponsiveness of T cells, a process called anergy (Mahnke et al., 2002), altered costimulatory molecule expression (Bakdash et al., 2013), or increased expansion and/or differentiation of Tregs (Sakaguchi et al., 2008). Because of their capacity to modulate autoreactive responses by inducing T cell anergy and regulatory Th polarization profiles, the use of DCs for immunotherapy has become an attractive possibility for treating autoimmune disease (Van Brussel et al., 2014). Different strategies have been used to generate tolerogenic DCs (tolDCs), such as pharmacologic intervention, biological agents, and genetic engineering (Nikolic and Roep, 2013; Everts and Pearce, 2014; Van Brussel et al., 2014). Pharmacologic intervention, including using vitamin D3, dexamethasone, aspirin, simvastatin, and rapamycin to generate tolDCs, could be achieved mainly by reducing DC surface expression of MHC molecules and costimulatory molecules. In contrast, biological agents, such as IL‐10, TGF‐β, and granulocyte colony‐stimulating factor, induce tolDCs by producing high amounts of anti‐inflammatory cytokines. On the other hand, advances in microRNA (miRNA) technology lead to the generation of tolDCs by targeting miRNA such as miR23b and small interfering RNA (siRNA) to silence NF‐κB or IL‐12. A recent study demonstrated that tolDCs induced by 1, 25(OH)_2_D_3_ (VitD3) significantly reduced the severity of EAE. The data suggest that tolDCs result in the inhibition of encephalitogenic T cell development through enhancing Tregs (Farias et al., 2013). Furthermore, Mansilla et al. (2015) found that treatment with VitD3‐generated tolDCs loaded with MOG_40–55_ peptide showed a beneficial effect in EAE, decreasing the incidence and clinical severity of disease while inducing the abundance of Tregs and IL‐10. To test whether tolDCs can block EAE development, Zhou et al. (2014) treated bone marrow–derived DCs with LPS and transferred DCs into EAE mice. The results showed that LPS treatment modulates the phenotype of DCs and generates tolDCs to block CD4^+^ T cells' activity, thus inhibiting the development of EAE in vivo. These results in a preclinical model can be considered as a proof of concept that tolDCs could be a potential specific cell‐based immunotherapy for MS. Giannoukakis et al. (2011) recently demonstrated the safety of tolDCs therapy in the first clinical trial using tolDCs in type 1 diabetes. Furthermore, Thomas et al. demonstrated the safety and biological activity of a single intradermal injection of autologous modified DCs exposed to citrullinated peptides in a phase 1 trial in patients with rheumatoid arthritis, which was reported in 2015 (Benham et al., 2015). Although using tolDCs as a therapeutic tool seems to be a promising therapeutic strategy for restoring tolerance in MS, there are many challenges to be faced from bench to bedside, such as choice and loading of autoantigens; type and source of DC (donor or recipient); the permanent ability to regulate the autoimmune response; tolerogenicity and specificity; identification of optimal combination regimens (with other immunosuppressive or tolerogenic strategies); overcoming late graft rejection; and immunologic memory. Further studies are needed before the clinical application of tolDCs in MS.

## STEM CELL THERAPY IN MS

While current therapies for MS are either immunomodulatory or immunosuppressive, those treatments reduce the frequency of relapses of the disease but do not prevent long‐term disease progression. With the recent progress in stem cells, therapies that aim at a combination of effective immunomodulation, or restoration of self‐tolerance and neuroprotection, should be taken into consideration. Originating in either embryonic or adult sites, stem cells are characterized by their ability to self‐renew and differentiate into multiple lineages, thereby contributing to the maintenance and repair of organs or tissues (Payne et al., 2011). Moreover, studies also showed that certain adult stem cell populations exert potentially beneficial immunomodulatory effects on CNS inflammation (Ben‐Hur, 2008; Caplan, 2009). There are various types of stem cells such as embryonic stem cells, HSCs, neural stem cells, mesenchymal stem cells (MSCs), and induced pluripotent stem cells (Xiao et al., 2015). Among the various types of stem cells, the therapeutic efficacy and safety of HSCs in the treatment of MS have been examined in some detail. Furthermore, recent burgeoning evidence of MSCs makes them a promising candidate for the treatment of MS.

### HSC Transplantation

HSCs constitute the main population of stem cells found in the bone marrow, giving rise to cells belonging to the myeloid and lymphoid lineages. Since the first study on successful HSC transplantation (HSCT) reported in the 1960s (Kondo et al., 2003), the procedure has become a widely used treatment option, principally for the treatment of hematologic malignancies, such as leukemia and lymphoma. And now HSCT is being investigated as a therapy for highly aggressive patients with malignant forms of MS who are unresponsive to conventional therapies (Mancardi and Saccardi, 2008). While conventional MS therapies rely on the mechanisms of immunomodulation and immunosuppression to inhibit the inflammatory process, the goal of applied HSCT is to completely remove all potentially autoreactive cells from the periphery and create a nonautoreactive immune environment. This procedure is believed to reset the immune system and induce a prolonged tolerance toward self‐antigens except for its certain immunosuppressive properties following the preparation of the recipient for the transplant (Muraro and Abrahamsson, 2010; Darlington et al., 2013; Muraro et al., 2014). More than 800 patients with MS have been treated worldwide with the procedure in recent years, and the treatment appears to be very effective compared with immunosuppression followed by mitoxantrone (MTX), especially in malignant MS cases (Mancardi et al., 2015). Recently, a small study using a high dose of an immunosuppressive compound followed by transplantation of patient‐derived HSCs resulted in overall event‐free survival of 78.4% (90% confidence interval [CI], 60.1%–89.0%) over 3 years. Progression‐free survival and clinical relapse‐free survival were 90.9% (90% CI, 73.7%–97.1%) and 86.3% (90% CI, 68.1%–94.5%), respectively, during 3 years (Nash et al., 2015). Patients also showed improvements in neurologic disability, quality of life, and functional scores (Nash et al., 2015). Mancardi et al. (2015) reported the first randomized controlled multicenter trial of intense HSCT vs. MTX for treatment of MS. The disease activity was evaluated by magnetic resonance imaging (MRI). The main findings were that HSCT reduced by 79% the number of new T2 lesions compared with MTX in the 4 years following randomization. And it also reduced gadolinium‐enhancing lesions as well as the annualized relapse rate. However, no difference was found in the progression of disability. Despite some evidence demonstrating the efficacy of HSCT, several issues associated with HSCT need to be taken into consideration. Patients can experience transient neurological deterioration, other toxicity‐related infection, allergic reactions and even the progression of brain atrophy after transplantation (Payne et al., 2011). Further refinement is clearly needed to ensure that treatment benefits outweigh the associated risks.

### MSC Transplantation

MSCs can be isolated from numerous tissue sources such as bone marrow, amniotic fluid, dental pulp, adipose tissue, umbilical cord, synovial membranes, and peripheral blood, but most studies focused on bone marrow derived–MSCs (Xiao et al., 2015). Recently, the immunomodulatory and immunosuppressive properties of MSCs have been well established. In 2005, Zappia et al. (2005) showed that the intravenous injection of MSCs improved the clinical signs of the EAE induced by MOG and reduced demyelination and leukocyte infiltration in the CNS. MSCs can cause T cell anergy, since T cells isolated from the lymph nodes of MSC‐treated mice did not proliferate in vitro after the exposure to new MOG peptide. This result suggests that MSCs can induce peripheral immune tolerance. After that, Kassis et al. (2008) found that an improvement of the clinical signs and a reduction of inflammation in the CNS, accompanied by significant protection of axons, were observed in MSC‐treated EAE. These results indicate that MSCs may provide a new way to provide neuroprotection and immunomodulation, and possibly to promote remyelination and neuroregeneration in EAE. Recent studies have also shown that MSCs might have the capacity to reduce immune attacks in patients with MS. Karussis et al. (2010) have demonstrated that MSCs can be safely used, creating the possibility to design future efficacy trials in patients with active MS. There are many issues that need to be taken into consideration, including cell number, frequency, and duration of administration. In a randomized, placebo‐controlled, phase 2 trial, 9 patients with relapsing–remitting MS received intravenous infusion of bone marrow–derived MSCs for 6 months (Llufriu et al., 2014). The results indicated that the patients treated with MSCs had a trend to lower mean cumulative number of gadolinium‐enhancing lesions on MRI, and a decrease of the frequency of Th1 (CD4^+^ IFN‐γ^+^) cells in the blood of MSC‐treated patients was observed. There were no delayed adverse event reports after completion of the 12‐month protocol. In another open‐label phase 2a trial designed to assess the safety and efficacy of autologous MSCs as a feasible neuroprotective treatment for secondary progressive multiple sclerosis (SPMS), 10 patients with SPMS involving the visual pathways were recruited and received intravenous infusion of autologous bone marrow–derived MSCs. After treatment, an increase in optic nerve area and reduction in visual evoked response latency without evidence of significant adverse events were observed (Connick et al., 2012). These findings are consistent with the neuroprotective effect of MSCs by stopping the autoimmune attack against myelin antigens and promoting nervous tissue repair. Therapeutic application of MSCs for treatment of MS may thus provide a method of promoting neuroprotection and neuroregeneration.

## CONCLUSION

It has been clearly documented that many immune cell populations are involved in MS and EAE, some playing pathogenic and others playing protective roles during various phases of disease. Cell‐based therapies for the treatment of MS and EAE have thus been proposed. Here we summarize recent studies that show that cell‐based therapies produced a variety of biological effects in MS and EAE, attenuating disease severity and inflammation in the CNS. The data presented here are limited, and the use of cell‐based therapies is still in its infancy. There are many issues that need further exploration, including optimal purification and ex vivo amplification, the most safe and efficacious route, cell number, and frequency and duration of administration.

## CONFLICT OF INTEREST STATEMENT

The authors report no conflicts of interest.

## ROLE OF AUTHORS

Primary authorship: Y.C., L.S. Significant contributing authorship and editing of intellectual content: Z.X., X.F., Q.Q.C. Critical review and editing of intellectual content: J.H., J.Z. Study supervision: T.J.
